# A Seamless Ubiquitous Telehealthcare Tunnel

**DOI:** 10.3390/ijerph10083246

**Published:** 2013-08-02

**Authors:** Po-Hsun Cheng, Bor-Shing Lin, Chu Yu, Shun-Hsiang Hu, Sao-Jie Chen

**Affiliations:** 1 Department of Software Engineering, National Kaohsiung Normal University, No. 62, Shenjhong Road, Yanchao District, Kaohsiung 82444, Taiwan; 2 Department of Computer Science and Information Engineering, National Taipei University, No. 151, University Road, Sanshia District, New Taipei 23741, Taiwan; E-Mail: bslin@mail.ntpu.edu.tw; 3 Department of Electronic Engineering, National Ilan University, No. 1, Sec. 1, Shenlung Road, Yilan 260, Taiwan; E-Mail: chu@niu.edu.tw; 4 Graduate Institute of Electronics Engineering, National Taiwan University, No. 1, Sec. 4, Roosevelt Road, Taipei 10617, Taiwan; E-Mails: agg2567@gmail.com (S.-H.H.); csj@ntu.edu.tw (S.-J.C.)

**Keywords:** communication tunnel, mobile device, stream control transmission protocol, telemedicine, ubiquitous computing

## Abstract

Mobile handheld devices are rapidly using to implement healthcare services around the World. Fundamentally, these services utilize telemedicine technologies. A disconnection of a mobile telemedicine system usually results in an interruption, which is embarrassing, and reconnection is necessary during the communication session. In this study, the Stream Control Transmission Protocol (SCTP) is adopted to build a stable session tunnel to guarantee seamless switching among heterogeneous wireless communication standards, such as Wi-Fi and 3G. This arrangement means that the telemedicine devices will not be limited by a fixed wireless connection and can switch to a better wireless channel if necessary. The tunnel can transmit plain text, binary data, and video streams. According to the evaluation of the proposed software-based SCTP-Tunnel middleware shown, the performance is lower than anticipated and is slightly slower than a fixed connection. However, the transmission throughput is still acceptable for healthcare professionals in a healthcare enterprise or home care site. It is necessary to build more heterogeneous wireless protocols into the proposed tunnel-switching scheme to support all possible communication protocols. In addition, SCTP is another good choice for promoting communication in telemedicine and healthcare fields.

## 1. Introduction

Usually, Mobile Nursing Carts (MNC) [1,2,3] used in the healthcare field require an uninterrupted wireless access mechanism. Since the wireless web-based applications might switch among diverse wireless communication standards, a design for a one-to-many switching tunnel is necessary. Such tunnel switching can be achieved by the software-defined radio concept. That is, the software-defined radio can replace the physical hardware and provide a seamless communication service in an omnipresent environment.

Most healthcare services are not interruptible when serving patients [1]. For example, the inpatient MNC is a movable piece of equipment that includes a notebook computer, portable medical instruments, and required care appliances [2]. The MNC is pushed by a nurse between bedsides and utilizes the wireless communication functionality of the notebook computer to access electronic health records from back-end medical databases. Although such an inpatient region is covered by the wireless communication environment, it is unavoidable for the MNC to occasionally lose communication connection if there are dead spaces in the original wireless access point [3].

To improve the quality of the medical care and to provide complete information to the healthcare staff, the hospital is required to collect a large amount of clinical data. Mostly, the hospital uses MNC to collect clinical data via Wi-Fi networks and to save the data to the server side. Such a service is available for disease tracking and for notifying healthcare staff when the patient’s condition has changed. This service not only provides better medical care but also enhances staff productivity. Therefore, a ubiquitous communication service would be welcome in several industrial fields, such as transportation [4,5], manufacturing [6], and military [7,8]. Moreover, these healthcare-related examples can be found in some reviews [9] and forecasting [10] articles.

The coverage rate of Wi-Fi is often limited [2,3], because the signal dead ends will result in wireless disconnection. This problem can be solved by switching to a channel that has a better signal strength, say 3G. During the switching, the physical connection must first be disconnected and then reconnected again. It is therefore necessary to design and develop a seamless communication switching mechanism among available wireless access points to keep the original session alive and to facilitate uninterrupted healthcare service. It is interesting to design a mechanism to keep the logical connection continuous. In this study, tunnel middleware using the Stream Control Transmission Protocol (SCTP) [11] is proposed to solve the above-mentioned problem. The subsections that follow introduce the required information technologies, such as session control and SCTP in detail.

### 1.1. Session Control

Typically, we can achieve session control in an application layer, a transport layer, or a network layer. The details are described below.

1. *Application Layer*: A system can use a session control unit in the application that must maintain the same session. If a data access problem exists, then the system can add cookies to solve it. That is, the system can hold the same session while changing the Internet Protocol (IP), through the session control unit. The system also provides data integrity, requesting a server to resend data automatically when the data is incomplete. The advantages include at least that the change in the system environment is small, no extra network device is required, and the system implementation is simple. In contrast, the drawbacks usually include that the application needs redevelopment, the cookie style development might be necessary, and the complexity for application redevelopment is increased.
2. *Transport Layer*: In 2000, the Internet Engineering Task Force (IETF) defined a new transport layer protocol, SCTP, for Internet phone and video utilization. This protocol combines the advantage of the Transmission Control Protocol (TCP) [12] and the User Datagram Protocol (UDP) [13] as well as a multi-homing mechanism to enable a mobile device changing the IP without affecting the data transmission. The advantages include that the cost is smaller than the mobile IP solution and that it is not necessary to change the network’s physical architecture. However, the major disadvantages are that the Internet is TCP-based and most Hypertext Transfer Protocol (HTTP) and File Transfer Protocol (FTP) applications cannot be directly executed in the SCTP environment.
3. *Network Layer*: The development of a mobile network with mobile devices is popular on the Internet. However, the IP layer is not designed for the mobile devices, and the support of the IP for mobile devices is relatively weak. Therefore, the IP cannot satisfy the high-mobility requirements of these devices. For this reason, a “mobile IP” [14] is needed and was designed for solving the IP switching problem in the network layer. The advantages for using a mobile IP include compatibility with other network services, good security, and scalability. In contrast, the shortcomings include the need for a high-cost network device, the inability to support both IPv4 and IPv6, low flexibility for switching when the IP is changed, and that the IP switching time might exceed the TCP [12] timeout constraint.

### 1.2. SCTP

The SCTP is a reliable message-oriented transport layer protocol [12,14,15,16,17]. The following outline describes the services that are provided by the SCTP.

1. *Process-to-Process Communication*: Similar to the TCP and UDP mechanisms, the SCTP provides process-to-process communication and can replace all applications that are TCP-based originally [14]. On the other hand, there are some applications that have been developed for SCTP utilization, such as the Integrated Services Digital Network User Adoption (IUA) [18], the Message Transfer Part Level 2 for User Adoption (M2UA) [19], the Message Transfer Part Level 3 for User Adoption (M3UA) [20,21], the H.248 [22] Gateway Control Protocol, the H.323 [23] Packet-based Multimedia Communication Systems, and the Session Initiation Protocol (SIP) [24].
2. *Connection-Oriented Service*: Similar to the TCP service, the SCTP is a connection-oriented protocol, also. However, every connection in the SCTP service is referred to as an association [14].
3. *Reliable Service*: Similar to the TCP service, the SCTP is a reliable transport layer protocol. The SCTP checks the destination of every packet [14].
4. *Multiple Streams*: In the TCP mechanism, there is a single stream between client and server. This stream is one of the connection bottlenecks of the TCP. Furthermore, the TCP session might be disconnected, and data will be lost in the case of a client or server connection error. If such an error occurs in a text transmission service, the effects could be tolerable. However, it might be a larger problem for instant data transmission such as video or voice transmission. Correspondingly, the SCTP provides multiple streams with an association. The multiple streams in the association will not affect the data transmission, even if one of the streams receives a connection error [14].
5. *Multi-Homing*: In a TCP session, there is a unique destination IP and source IP pair. If the device has multiple network interfaces and IPs, it can choose only one IP and interface to connect with the server. However, in an SCTP session, the multi-homing mechanism can define more than one IP in an association. The data transmission is not affected, even if one of the interfaces crashes. This mechanism is very important, especially in the instant data transmission service [14].

Table 1 shows the relationship among the SCTP and other protocols in the Open Systems Interconnection (OSI) model and the Department of Defense (DoD) model. Note that a few of the network applications are designed by the SCTP. Therefore, most of the network applications must be redeveloped when the SCTP mechanism is used. However, redevelopment work is difficult and costly because designers must carefully process their tasks simultaneously among several layers.

**Table 1:** Relationship among SCTP and other protocols.

Layer	OSI Model	DoD Model	Protocols
Layer 7	Application	Process, Application	HTTP, FTP, Secure Shell (SSH), Post Office Protocol (POP), Simple Mail Transfer Protocol (SMTP), Domain Name System (DNS), Health Level Seven (HL7), Digital Imaging and Communications in Medicine (DICOM)
Layer 6	Presentation		
Layer 5	Session		Network File System (NFS), Server Message Block (SMB), Socket Secure (SOCKS)
Layer 4	Transport	Host-to-Host	TCP, UDP, SCTP
Layer 3	Network	Internet	IPv4, IPv6, Internet Protocol Security (IPSec), Address Resolution Protocol (ARP), Internet Control Message Protocol (ICMP)
Layer 2	Data Link	Network Access	Point-to-point Protocol (PPP), Fiber Distributed Data Interface (FDDI)
Layer 1	Physical		RS-232, Universal Serial Bus (USB), Asymmetric Digital Subscriber Line (ADSL), Integrated Service Digital Network (ISDN)

Based on the above technology, a large number of studies have reported and obtained related outcomes. This study therefore attempts to design and implement the required seamless communication mechanism for use in the ubiquitous healthcare field. The following section delineates our design and the experimental results.

## 2. Design and Implementation

This study proposes a software-based SCTP-Tunnel middleware. The proposed middleware builds a tunnel between the client and server sides to solve the TCP disconnection problem by carrying the TCP packets on the SCTP streams. A SCTP packet filter can catch the TCP packets, then pack them into SCTP packets, parse the TCP packets from the SCTP packets, and send them to the destination server via the same tunnel. This system can even reply the TCP acknowledge packet via the tunnel. The proposed SCTP-Tunnel middleware is compatible with IPv4 and IPv6, and it is not necessary to modify any applications. This design utilizes the SCTP packet acknowledge and multi-homing mechanisms to achieve network fault tolerance.

Most Hospital Information Systems (HIS) are TCP-based. It is therefore predictable that there is a large bottleneck for a session control service. Even if the engineers use mobile IP or redevelop applications for changing the TCP to the SCTP, the cost is high. Our SCTP-Tunnel does not need to change the HIS codes. Meanwhile, the SCTP-Tunnel can process session control and is compatible with the TCP mechanism.

**Figure 1:**
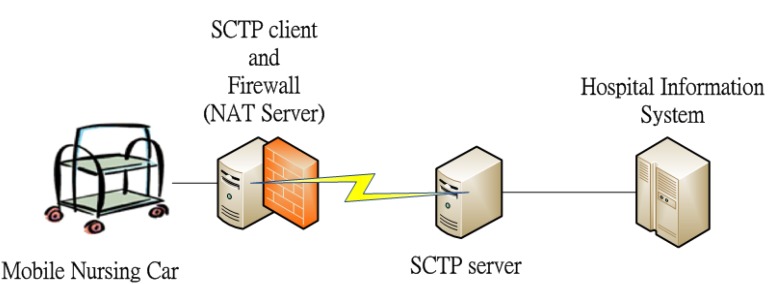
Deployment diagram of the SCTP-Tunnel middleware.

Figure 1 shows the deployment diagram of the proposed SCTP-Tunnel middleware. This system can be divided into two distinct parts: the SCTP client and the SCTP server. First, the SCTP client can create an SCTP tunnel with the SCTP server when the MNC is sending data to the HIS. Basically, the SCTP client will block the packets through a firewall, pack the TCP packets into the SCTP packets, and send the SCTP packets to the SCTP server through the SCTP tunnel. Second, the SCTP server will get the SCTP packets transmitted by MNC, revert back from the SCTP packets to the TCP packets, and edit the TCP packet headers. Third, the SCTP server will transmit the new TCP packets to the HIS.

Moreover, the existing SCTP*-*compatible kits must be installed in the mobile device, and the deployment is unfriendly for the engineers and caregivers. Hence, this study improves this weak point by adding an Internet box that includes an SCTP client to provide Wi-Fi and 3G services. The SCTP client can also create an SCTP-Tunnel with the SCTP server automatically to achieve session control.

### 2.1. Experimental Environment

First, the SCTP server is running on the Ubuntu v12.04 operating system. The proposed design sets it to stop running the system only if the TCP packet’s header flag is RST (reset) through the “iptables” [25]. Meanwhile, the SCTP client’s operating system is also running on Ubuntu v12.04.

Within the implementation period, this work had been tested by the SCTP server on the FreeBSD v8.0 operating system. However, a fatal error was encountered, which presented as “kern/167217: catch an over 1,024 byte network packet through libpcap in C.” Such an error message was reported to the FreeBSD maintenance team; the FreeBSD team has confirmed that it is a system bug. Therefore, this work decided to port the source code from FreeBSD to Ubuntu. The deployed SCTP-Tunnel server has been proven for its practicability.

### 2.2. SCTP-Tunnel Middleware

**Figure 2:**
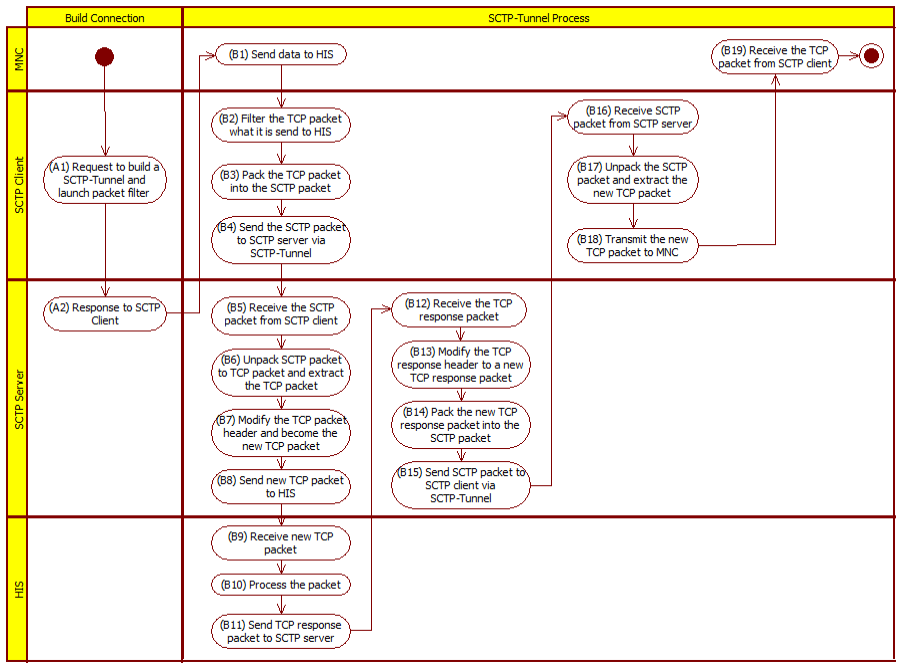
Activity diagram for the SCTP-Tunnel middleware.

The activity diagram of the SCTP-Tunnel middleware is shown in Figure 2. Note that the SCTP client will be embedded at the top of the MNC and connected with the notebook at the top of the MNC through an RJ-45 cable. Therefore, the SCTP client provides a network connection to the MNC and decides to transmit packets to the Internet through either a Wi-Fi or a 3G communication channel.

First, the SCTP client creates an SCTP-Tunnel with the SCTP server such that the client can send packets to the SCTP server through the SCTP-Tunnel. After the SCTP-Tunnel is constructed completely, the SCTP client can provide service to keep the session alive and continuous. These processes are shown as the A1 and A2 steps at the top of Figure 2.

As shown in Figure 2, there are nine steps for the MNC to transmit data to the HIS. The detailed procedures are described as followings: when MNC sends specific data to HIS, the TCP packet will transmit it to the SCTP client and the SCTP client will pack all of the TCP packets into SCTP packet(s) and send the SCTP packet(s) to the SCTP server through the SCTP-Tunnel. After the SCTP server gets the SCTP packet(s), the SCTP server will unpack the SCTP packet(s) and extract the TCP packet sent by MNC. It will also change the TCP packet’s IP header from the source IP into the IP of the SCTP server and make it a new TCP packet. Then, the SCTP server sends the new TCP packet to the HIS through the raw socket mechanism.

**Figure 3:**
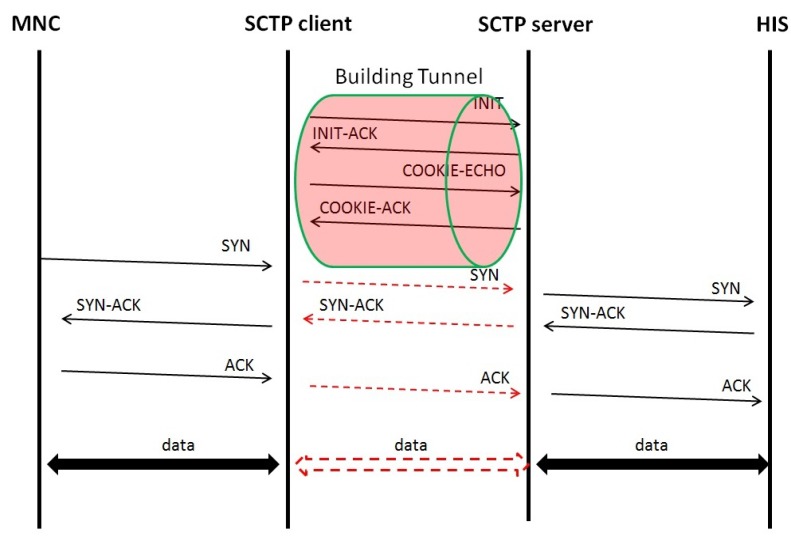
TCP three-way handshaking in the SCTP-Tunnel.

After the HIS receives the new TCP packet, a necessary process will be initiated and a response TCP packet will be acknowledged to the host that is following the source IP of the new TCP packet with the SCTP server IP. Then, the SCTP server will receive a response TCP packet that comes from the HIS side. There are another nine steps for processing the acknowledge packet, which are shown in Figure 2. Next, the SCTP server will change the IP header from the destination IP to the IP of the MNC to become a new TCP packet. The SCTP server will pack all of the new TCP packets into SCTP packet(s) and send the SCTP packet(s) to the SCTP client through the SCTP-Tunnel. The SCTP client will receive the SCTP packet(s), unpack the SCTP packet(s), and extract the new TCP packet. Then, the SCTP client will send the new TCP packet to the MNC through the raw socket mechanism. The schematic diagram of achieving the TCP connection through the SCTP-Tunnel is shown in Figure 3. In such a way, the TCP three-way handshaking mechanism in the SCTP-Tunnel is implied in this design.

In addition, SCTP [26] is adopted inside this platform using the TCP and UDP advantages and supports a multi-homing function for network fault tolerance. An IP in IP technology is emerging to encapsulate one IP packet in another IP packet. That is, a packet reassembly mechanism is designed to cut the original TCP packet into a fixed size of 800 bytes per packet. Hence, two self-defined packets are proposed, as shown in Table 2.

**Table 2:** Format of self-defined SCTP packets.

**Information Header**	**Byte**	1	2	3	4	5
	**Data**	0xFF	IP Header ID1	IP Header ID2	Total Cut Number	SCTP Packet ID
**Switch IP**	**Byte**	1	2	3	4	5
	**Data**	0xFF	0xFF	0xFF	0xFF	0xFF

As designed, every SCTP packet owns two self-defined packets: Information Header and Switch IP. The former packet carries the required packet information, and the latter packet is utilized during the SCTP client to switch the IP from the original IP to a new IP. That is, the SCTP client will send this packet to the SCTP server and notify to start the IP switching process. After the SCTP server receives this packet, the subsequent packets will not transmit to the SCTP client’s original IP address and, instead, will transmit to a new IP address.

### 2.3. Revised SCTP Tunnel Middleware

In addition, this study also presents a revised version of the original one-to-one SCTP-Tunnel middleware. This middleware was implemented in the ANSI C language, and the related coding techniques were reported. The following sections describe the affiliated schemes, including the SCTP-Tunnel’s one-to-many mechanism, the Nursing Notes System (NNS), the decubitus ulcer risk assessment service, and the medical image browsing service.

#### 2.3.1. SCTP-Tunnel’s One-to-Many Mechanism

Initially in this research, we implemented the SCTP-Tunnel code for one-to-one service. The service is client/server-based software and can be executed on the Ubuntu v11.10 operating system. To enhance the SCTP-Tunnel service from a one-to-one to a one-to-many mechanism, the client and server codes were rewritten. According to our experience, the SOCK_STREAM and SOCK_SEQPACKET flags can be activated in the one-to-one and one-to-many mechanisms, respectively. Note that the SOCK_STREAM flag represents a reliable stream-oriented service (stream sockets), and the SOCK_SEQPACKET flag stands for a reliable sequenced packet service.

#### 2.3.2. Nursing Notes System

The NNS is a significant service for nurses, and it is also a compulsory tool for caregivers to provide high-quality healthcare service. To evaluate the communication performance of the text-based health records, the NNS offers a few major services, such as the Admission Nursing Care Assessment (ANCA), Family Tree (FT), Physical Assessment (PA), Decubitus Ulcer Risk Assessment (DURA), Fall Risk Assessment (FRA), Constraint Assessment (CA), and Medical Image Browsing (MIB). Two of these services, the DURA and the MIB, are described in the following paragraphs.

**Figure 4:**
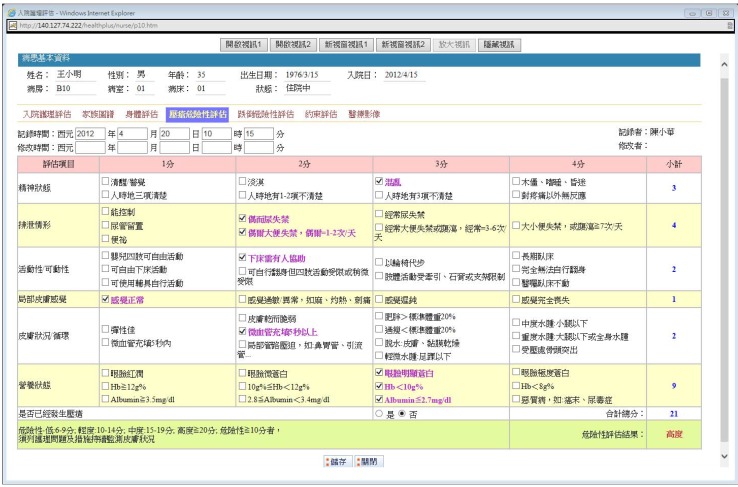
Decubitus ulcer’s risk assessment service.

First, the DURA service is shown in Figure 4 and supports a fillable form that the nurse can use to record the patient’s risk evaluation for decubitus ulcers. There are six categories, and the selected options can be checked by a caregiver. Then, the DURA calculates the assessment value automatically, and this value is shown at the bottom of the form. For example, the assessment value is 21 for the specific patient in Figure 4. That is, the patient is in the high-risk range (≥20). Lastly, the caregiver can save the assessment result by clicking the save button. Note that the size of the whole web page is approximately 25 KB.

**Figure 5:**
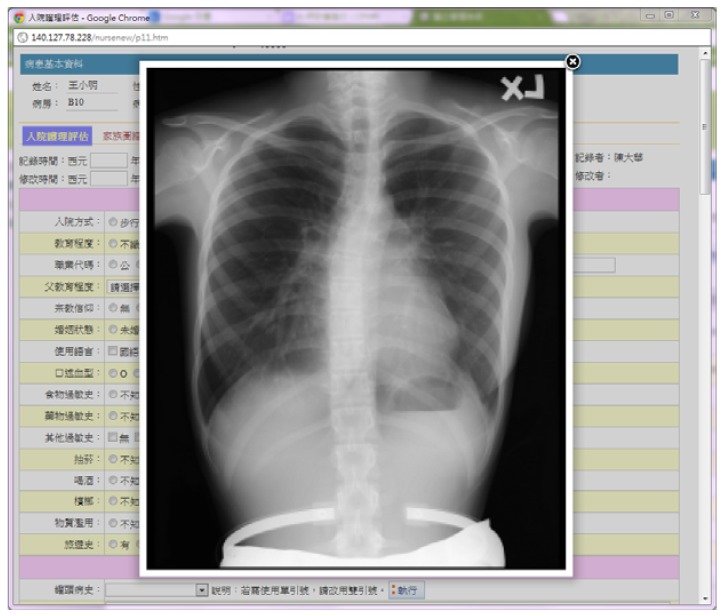
Medical image browsing service.

Second, the NNS also includes the MIB service to evaluate the communication performance of image-based health records. The MIB service is shown in Figure 5. The drop-down list box shows two types of experimental image sizes, such as 8 MB and 33 MB. Typically, the size of a single X-ray image stored in a bitmap format is approximately 8 MB. Note that the large medical image file is regenerated from the ordinary medical image file using a linear interpolation method. That is, a sizable medical image file can be used to evaluate the SCTP-Tunnel performance through the MIB service.

**Figure 6:**
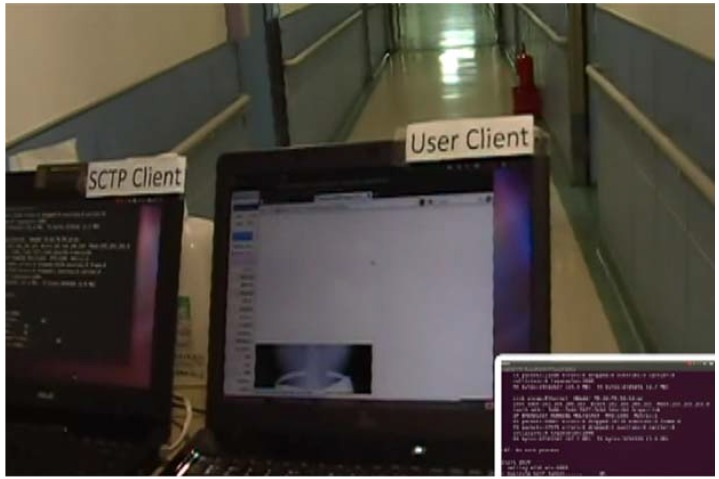
Seamless wireless switching between Wi-Fi and 3G in the hospital.

The most compelling evidence is that Figure 6 shows a smooth network switching scenario between Wi-Fi and 3G wireless channels in the hospital; additionally, the deployed platform can process large medical images and video conferences.

## 3. Results and Discussion

**Figure 7:**
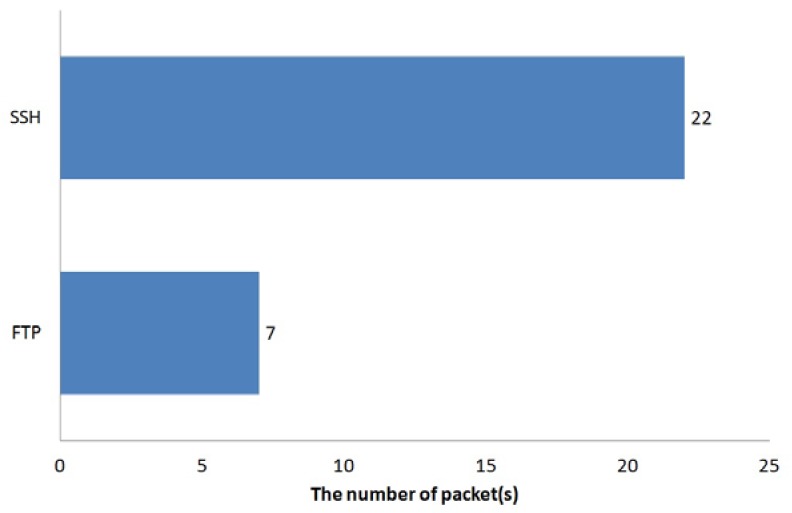
Numbers of packets used in creating the SSH and FTP connections.

**Figure 8:**
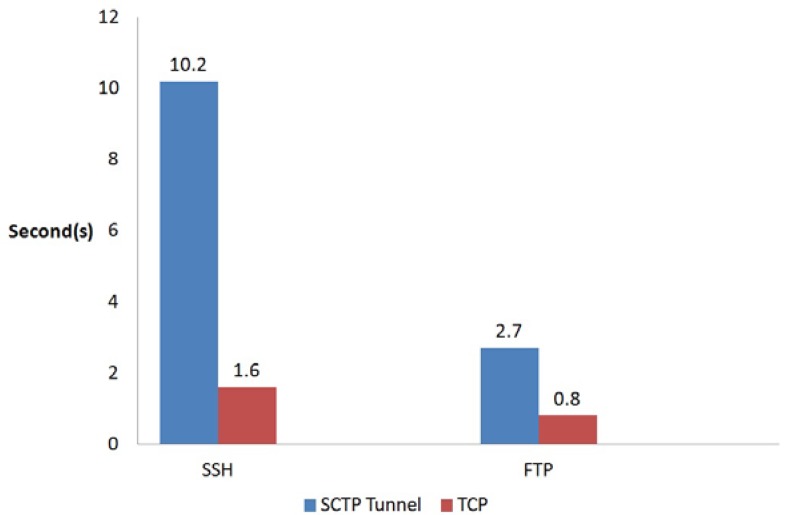
Time costs in creating the SSH and FTP connections.

It is evident that the proposed SCTP-Tunnel middleware utilizes additional processes for a packet, while it might increase the packet transmission time. The numbers of packets required in the experiments when creating the SSH and FTP connections are shown in Figure 7. Furthermore, Figure 8 illustrates the comparison of the time expenses in creating connections through the SCTP-Tunnel middleware and the original TCP mechanism. Through the analysis of the numbers of packets that were required to construct the SSH and FTP connections and the time costs in creating these two connections, the difference in the times required in the SCTP and FTP connections can be divided by the number of packets sent to obtain an average packet delay-time of 0.39 sec for the SSH connection and 0.27 sec for the FTP connection. Because the MNC needs to send only the patient data, there are not a large number of packets to send in a short time. Therefore, the delay time will not affect the caregiver’s operation in the healthcare enterprise.

**Figure 9:**
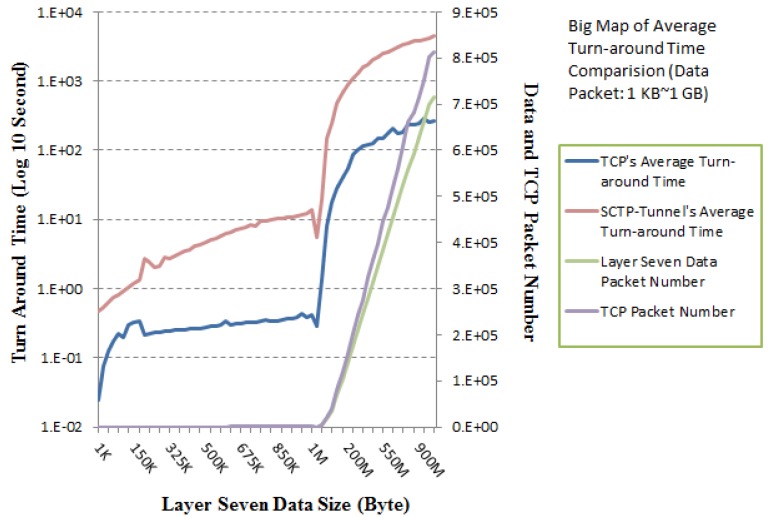
Turn-around time comparison.

To upgrade the proposed SCTP-Tunnel performance, a speculative measurement for data packets (1 KB*–*1 GB) is executed. The empirical result is shown in Figure 9, and it illustrates the juxtaposition of the turn-around time between the SCTP-Tunnel middleware and the original TCP. The metric scope does not cover the time expense of the TCP three-way handshaking process. At the same time, this work repeats the same measurement three times to request data from the server. Every request is stopped for one second after the previous transmission is completed. The test data sizes from layer seven (the application layer) range from 1 KB to 1 GB to satisfy the diverse transmission requirements for both healthcare text files and medical image files.

**Figure 10:**
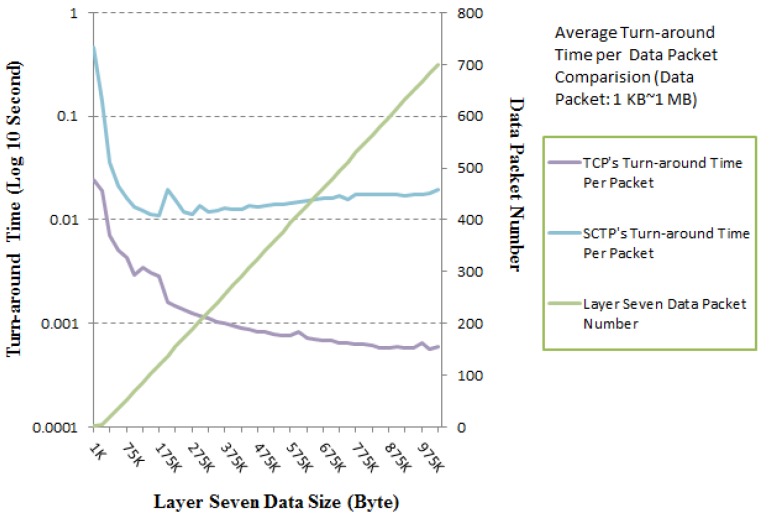
Turn-around time per packet comparison between TCP and SCTP-Tunnel for various data packet numbers: 1 KB–1 MB.

**Figure 11:**
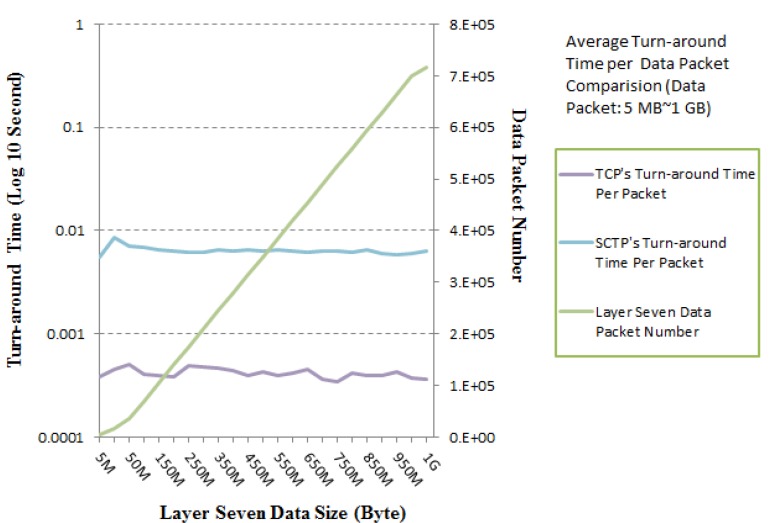
Turn-around time per packet comparison between TCP and SCTP-Tunnel for various data packet numbers: 5 MB–1 GB.

Figure 9 illustrates the turn-around time per data packet in layer seven. Note that the average turn-around times per data packet for the SCTP-Tunnel with small (1 KB–1 MB) and large (5 MB–1 GB) numbers of data packets are approximately 29.0050 ms and 6.4244 ms, respectively. The average turn-around times per data packet over TCP for small (1 KB*–*1 MB) and large (5 MB–1 GB) numbers of data packets approach 2.3310 ms and 0.4115 ms, respectively. These numbers are both reasonable average turn-around times per data packet. The performance test for the small number of data packets presents a rugged curve in Figure 10, because the total number of packets includes a relatively large number of three-way handshaking packets. Correspondingly, Figure 11 has a smoother curve.

In contrast, our suggested SCTP-Tunnel service is enacted in the nonspecific TCP environment. There are a number of interesting results that indicate the difference in the layer seven data packet numbers and the TCP packet numbers. For example, one layer-seven data packet requires at least eight TCP packets for transmission. That is, seven additional TCP packets contain three-way handshaking packets and acknowledgment packets for completing a successful communication. The distinctions between the layer-seven data and TCP packet numbers for small (1 KB*–*1 MB) and large (5 MB–1 GB) packet numbers are approximately 89.7674 and 52,069.9, respectively. Additionally, the average packet number differences per data size (KB) for small (1 KB*–*1 MB) and large (5 MB–1 GB) packet numbers are approximately 0.43977 and 0.10782, respectively. For a larger number of data transmission packets, a smaller number of extra control packets are required for TCP and the proposed SCTP-Tunnel service.

As noted before, this work designed an SCTP-Tunnel middleware that is working in an existing network environment and is compatible with the TCP mechanism. That is, using the SCTP-Tunnel, users can achieve a better connection than the TCP mechanism, as shown in this work. However, this approach still cannot judge the wireless transmission rates and select the optimal route from multiple routes. It is feasible to provide a tunnel service via the SCTP-Tunnel that connects to service indirectly to approach the Wi-Fi and 3G switching seamlessly, without changing the original system architecture. Such a service will also provide high quality in long-term healthcare services in healthcare enterprises.

While roaming among heterogeneous networks, the switching time is approximately 22–24 ms, which is shown in Table 3. Our approach can decrease the packet loss during the switching process. The SCTP-tunnel can resend the TCP packet and accomplish the process to maintain the original connection in case of packet loss. We can set the TCP window value to one and wait an extra time interval to let TCP detect the new network status and adjust the TCP window to the correct value.

**Table 3:** Average switching time between Wi-Fi and 3G networks.

	Test Count	10^5^ Times			10^6^ Times
Switch Direction		First Test	Second Test	Third Test	First Test
**From Wi-Fi to 3G**		22.958 ms	22.951 ms	23.037 ms	22.954 ms
**From 3G to Wi-Fi**		22.970 ms	22.961 ms	23.132 ms	22.894 ms
**Average Switching Time**		*22.964 ms*	*22.956 ms*	*23.084 ms*	*22.924 ms*

When MNC sends specific data to the HIS through the SCTP-Tunnel middleware, it will generate a slight delay. The delay might affect the efficiency of the MNC services slightly, but the advantage is that we can still provide a continuous connection when switching between 3G and Wi-Fi communication channels is needed. The SCTP-Tunnel middleware enables the MNC service to be more user-friendly and efficient. Hence, such middleware can be embedded in the required mobile devices. This work also adds features for live video transmission on HIS via WebRTC through which physicians can preview a specific patient’s condition before he/she enters the ward.

To sum up briefly, the proposed SCTP-Tunnel can be implemented as a reusable element for any operating system, is compatible with IPv4 and/or IPv6, and utilizes the SCTP packet acknowledge and multi-homing mechanisms to achieve network fault tolerance. The SCTP-Tunnel can be adopted without modifying the applications and can perform simultaneous session management with a seamless switching mechanism. The system is also convenient and has feasible execution performance for general healthcare services.

The experimental results of the one-to-many SCTP-Tunnel service illustrated a rational growth curve. Nevertheless, the observed SCTP-Tunnel performance is not higher than that of the TCP service. Considering that most health professionals are engaged in their duties, this design must improve the source code and provide them with better service quality. An on-line SCTP-Tunnel management panel must be designed and enforced to optimize the system parameters of the SCTP-Tunnel service.

The Network Address Translation (NAT) can support the SCTP protocol; however, at least three problems arise and are listed below:

1. The SCTP, TCP and UDP are not similar; for example, they differ in the packet format and the computational method for checksum. While the NAT is translating, it is necessary to modify the IP address and the checksum of the layer four packets. Hence, the NAT must support the SCTP packet format.
2. While the NAT is translating, some NATs cannot fully map the IP address. For example, the HostA transfers an “init” packet and passes it to the PEER side via the NAT. While the PEER side replies with an “init-ack” packet, it might not be transferred to the HostA via the NAT, and we lose the connection.
3. The SCTP owns a multi-homing function. While multiple networks are available, one is for a major route and the others are for alternative routes. If the major route fails to transfer packets, one of the alternative routes is utilized for further transmission. If this alternative route is built under the NAT, at least two situations will meet. One situation is that the host cannot obtain the physical IP address. The other phenomenon is that the host can obtain a physical IP address and the PEER side utilizes an alternative route, but the NAT does not own related IP mapping information.

Therefore, our designed applications, such as NNS, did not include any embedded IP address information in the context. It is necessary to consider the NAT status in the implementation phase for real applications. We suggest that the designers include the Session Traversal Utilities for NAT (STUN) mechanism to obtain physical IP address information and achieve the NAT traversal capability in cases when heavy traffic applications are implemented for using our SCTP-tunnel.

The goals of this study are to provide a flawless solution to professionals in the healthcare field and even to professionals outside of the healthcare industry. Therefore, a number of further designs are proposed to implement prevailing software components, such as IP interchanging, spectrum sensing, desirable spectrum selection, clinical biomedical signal transmission, and video conference communication. For example, the IP interchanging component would handle the layer-four processes. Spectrum sensing would provide the available wireless spectrum list and related information for further reference. The proposed spectrum selection mechanism would use a statistical method to propose a better spectrum to the caregiver’s device.

## 4. Conclusions

To enhance computer resource access from mobile devices, such as MNC in the healthcare field, it is necessary to provide a continuous wireless mechanism. The proposed SCTP-Tunnel middleware is a system component that can be integrated with electronic healthcare systems, such as discharge notes and nursing notes, and it can be ubiquitously utilized in the healthcare field. Accordingly, this central component is a major success factor for facilitating omnipresent electronic services in a healthcare environment. The middleware provides an SCTP-Tunnel to switch between Wi-Fi and 3G seamlessly and to connect to service indirectly without changing the original system architecture. Such a seamless connection mechanism supports qualified long-term healthcare services in a healthcare enterprise and can be adapted from healthcare enterprise to personal health monitoring. Additionally, it can be integrated with other mobile medical measurement devices (e.g., ear thermometer, weight scale, sphygmomanometer, blood glucose, and pulse oximeter) via Bluetooth.
